# Cortical Thickness Index and Canal Calcar Ratio: A Comparison of Proximal Femoral Fractures and Non-Fractured Femora in Octogenarians to Centenarians

**DOI:** 10.3390/jcm13040981

**Published:** 2024-02-08

**Authors:** Flurina Guyan, Estelle Gianduzzo, Manuel Waltenspül, Michael Dietrich, Method Kabelitz

**Affiliations:** Clinic for Orthopaedics, Hand Surgery and Trauma Surgery, Stadtspital Zürich, Tièchestrasse 99, 8037 Zürich, Switzerland; flurina.guyan@uzh.ch (F.G.); estelle.gianduzzo@uzh.ch (E.G.);

**Keywords:** cortical thickness index, canal calcar ratio, femoral neck fracture, trochanteric fracture, geriatric, fragility fracture

## Abstract

**Background:** The cortical thickness index (CTI) is a measure of bone quality and it correlates with the risk of proximal femoral fractures. The purpose of this study was to investigate the CTI in femoral neck, trochanteric fractures and non-fractured femora in geriatric patients and to determine whether there is a correlation between the CTI and the presence of a fracture. **Methods**: One hundred and fifty patients (fifty femoral neck- (FNFx), fifty trochanteric fractures (TFx) and fifty non-fractured (NFx)) with a mean age of 91 (range 80–104) years were included. Hip radiographs (antero-posterior (ap), lateral) were evaluated retrospectively. Measurements on the proximal femoral inner and outer cortices, including CTI and Dorr’s canal calcar ratio (CCR), were assessed for inter-observer reliability (ICC), differences of each fracture and correlation of parameters. **Results**: The mean ap CTI on the affected side was 0.43, 0.45 and 0.55 for FNFx, TFx and NFx, respectively. There was a significant difference of the ap CTI and CCR comparing the injured and healthy side for both fracture cohorts (*p* < 0.001). Patients with FNFx or TFx had significantly lower CTI on both sides compared to the NFx group (*p* < 0.05). There was no difference for CTI (*p* = 0.527) or CCR (*p* = 0.291) when comparing both sides in the NFx group. The mean inter-observer reliability was good to excellent (ICC 0.88). **Conclusions**: In proximal femoral fractures, the CTI and CCR are reduced compared with those in non-fractured femora. Both parameters are reliable and show a good correlation in geriatric patients. Therefore, especially for geriatric patients, the CTI and CCR may help to predict fracture risk and consult patients in daily practice.

## 1. Introduction

Proximal femur fracture is a common injury in the geriatric, with a rising incidence over the past and future decades [1]. In Europe, the lifetime risk of hip fracture is postulated to be as high as 23% in women and 11% in men [2,3,4]. The consequences of proximal femoral fractures significantly affect the quality of life and function of older people [5]. Furthermore, proximal femoral fractures are associated with a cumulative mortality rate of 33% in one year [6,7] and cause high costs for the health care system [8]. Many non-modifiable factors are involved in the pathomechanism of fractures, such as high or increasing age, female gender and other factors [3,9,10]. In contrast, low-energy trauma, such as falling, is considered to be a partially modifiable risk factor for hip fractures [11]. In particular, with reduced bone quality, fractures occur during simple falls [12,13]. Reduced bone mineral density (BMD) belongs to the definition of osteoporosis [14]. The World Health Organization (WHO) defines osteoporosis with a BMD-T score greater than 2.5 standard deviations (SDs) below the population mean of young adults, which is currently determined using dual-energy X-ray absorptiometry (DEXA). The correlation between BMD of the femur and radiological indices, such as the cortical thickness index CTI, have already been studied [11,12,15]. Previous studies showed that a low CTI correlates with a low quantitative BMD [16]. Another parameter that has been studied is the canal calcar ratio (CCR); however, data for the geriatric patients are scarce in the literature [17,18].

Therefore, the main goal of this study was to investigate whether there is a correlation between the CTI and CCR in patients aged 80 years or more with a femoral neck or trochanteric fracture. Secondly, we hypothesized that affected femora show a smaller CTI and CCR than the femora without a fracture in geriatric patients. Furthermore, an analyzation of gender-specific differences was performed.

## 2. Materials and Methods

The electronic local database from 2016 to 2021 at our institute was reviewed to identify patients that met the following inclusion criteria: age of 80 years and older, trochanteric (*n* = 50) or femoral neck (*n* = 50) fracture due to a low-energy trauma, absence of prior surgery of the affected proximal femur, absence of signs for pathological fracture or malignant diseases, measurable contralateral femoral bone and existence of preoperative plain radiographs of the hip (antero-posterior (ap) and lateral/axial). For the control group (*n* = 50), it was assured that no fracture of the proximal femur of either side was present. Patients’ characteristics and information on surgical interventions were retrieved from electronic medical records. Approval from the local ethics committee and informed patient consent were obtained (BASEC number 2022-01961). 

**Figure 1:**
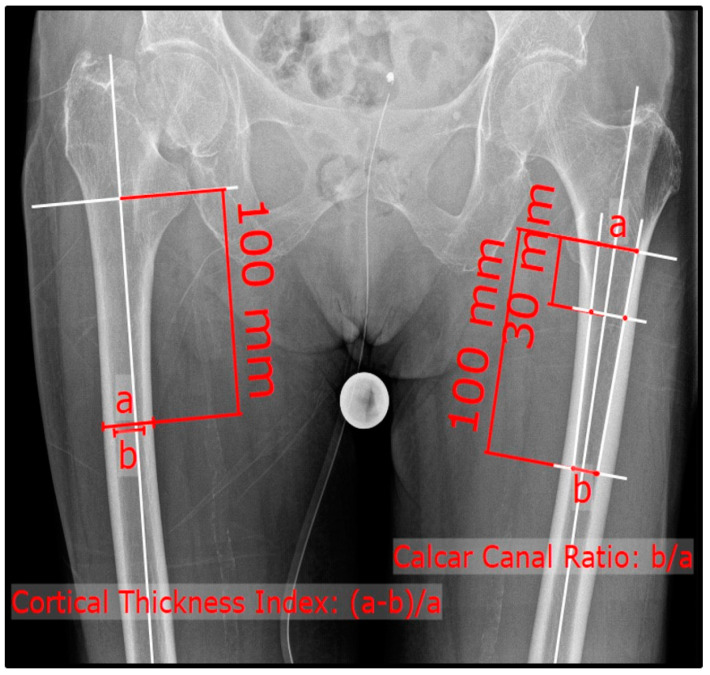
Antero-posterior X-ray of the pelvis; formula for the cortical thickness index (**right**) and canal calcar ratio (**left**).

**Figure 2:**
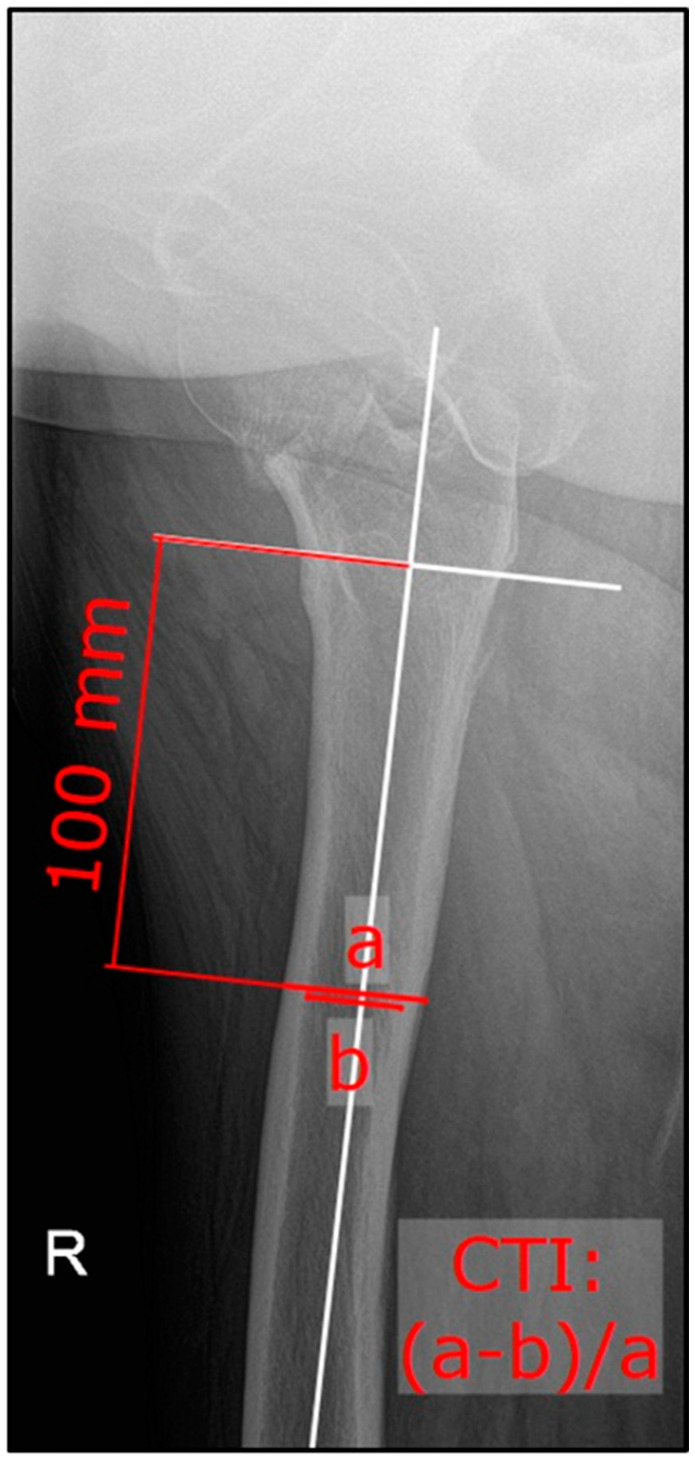
Lateral/axial X-ray of right hip; formula for the lateral cortical thickness index.

The patient was positioned supine on the radiographic table. The responsible radiology assistant performing the radiograph ensured an internally rotated position (15°) of the lower extremity if possible. The X-ray beam was centered on the symphysis. For the lateral/axial radiographs, the beam path was perpendicular to the axis of rotation of the selected leg, with the patient in a supine position and the contralateral hip flexed 90° and slightly abducted (45°). Measurements of CTI and CCR were performed according to the previously described methods using plain ap and lateral radiographs (Figure 1 and Figure 2) [19,20]. The ap CTI was calculated out of the outer and inner diameter of the femoral diaphysis ten centimeters below the lesser trochanter. The CCR depicts the quotient of the inner femoral diameter ten centimeters below the lesser trochanter to the inner distance of two lines, which are connecting the inner cortical points at three and ten centimeters below the lesser trochanter (Figure 1). In contrast, the lateral CTI was generated after measuring the outer and inner diameter in the lateral view (Figure 2). The CTI and CCR were measured bilaterally in the ap view and on the affected side (lateral X-ray). In the non-fractured group, the existing lateral radiograph was used. Measurements were performed using mediCAD clinical software Version 6.5 (mediCAD Hectec GmbH, Altdorf, Germany). 

To ensure inter-observer reliability, measurements were performed by three independent blinded observers (E.G., F.G. and M.K.), with two different levels of clinical experience (medical student, orthopedic consultant). 

To examine the gender-specific differences of the CTI and CCR a subgroup analysis for all three cohorts was performed.

### Statistical Analysis

In an a priori power analysis, 41 patients in each group were estimated to detect a difference of 0.05 with a power of 80% and an α of 0.05 based on the published means and standard deviations of the CTI using the G*Power tool (version 3.1; University of Düsseldorf Germany) [21]. Clinical characteristics of patients were determined using descriptive statistics. All data were assessed for normality using the Shapiro–Wilk test. Comparison of the injured proximal femoral with the healthy side was performed using paired *t*-test (normal distribution) or Wilcoxon signed-rank test (non-normal distribution). The independent *t* test (normal distribution) and Mann–Whitney U test (non-normal distribution) were used to compare continuous variables of the different groups. Pearson’s correlation was used to assess the relationship between radiographic measurements. According to Cohen’s assessment, a correlation of r > 0.5 is considered strong, an r value of 0.5–0.3 is considered moderate, and an r value of r < 0.3 is considered weak [22]. Interobserver reliability was assessed for CTI and CCR using the intraclass correlation coefficient (ICC) and the associated 95% confidence interval (95% CI). According to Landis and Koch, an ICC > 0.8 is considered almost perfect and an ICC between 0.8 and 0.6 is considered substantial [20]. All statistical analyses were performed using SPSS for Mac (version 23.0, SPSS Inc., Chicago, IL, USA). Significance was set at *p* < 0.05.

## 3. Results

### 3.1. Patient Demographic Characteristics

For the final analysis, 150 patients (50 femoral neck fractures (FNFx), 50 trochanteric fractures (TFx) and 50 non-fractured (NFx)) with a mean age of 92 (range 80–104) years were included. The vast majority of the patients were self-ambulatory prior to the trauma with a few individuals needing walking aids. Due to dementia or cognitive disorders, reliable information on the level of functionality in some patients was lacking. The patients’ demographic characteristics of the sub-groups are shown in Table 1.

### 3.2. Inter-Rater Reliability

The overall mean inter-rater reliability was good to excellent with an ICC of 0.88. Additionally, the group-specific ICC showed good to excellent results with an ICC of 0.85 ± 0.08 (range 0.77–0.94) for the FNFx-, 0.884 ± 0.03 (0.85–0.93) for the TFx- and 0.918 ± 0.04 (0.92–0.96) for the NFx cohort.

### 3.3. Comparison of CTI and CCR within the Sub-Groups

The mean ap CTI scores for FNFx and TFx were 0.434 ± 0.08 and 0.451 ± 0.09, respectively. Both parameters showed significant differences from the non-fractured side (*p* < 0.001). Similar results were observed for the CCR, whereas both cohorts with fractures (FNFx 0.735 ± 0.11, TFx 0.770 ± 0.17) presented a significantly higher value (*p* < 0.001). Table 2 provides an overview of the mean ap and lateral CTI and CCR of all sub-groups, showing that there are significant differences between the fractured and unfractured side in both groups with fractures. There was also a significant difference between ap CTI (*p* = 0.003) and the CCR (*p* = 0.016) of the non-fractured side of the FNFx group and the control group. Furthermore, a significant difference was found when comparing the mean ap CTI of the fractured site in the TFx group with that of the control group (*p* < 0.001). Additionally, the same comparison in each group’s contralateral side showed a significant difference (*p* = 0.018). The comparison of the mean CCR in the affected site of the TFx group with the equivalent of the control group showed a significant difference (*p* < 0.001), and the mean CCR comparison on both contralateral sites also showed a significant difference (*p* < 0.002). For both groups with fractures, the mean lateral CTI showed significantly smaller values than the individuals with no fractures (FNFx 0.417 ± 0.07 vs. NFx 0.458 ± 0.08, *p* = 0.008; TFx 0.416 ± 0.06 vs. NFx 0.458 ± 0.08, *p* = 0.002). 

### 3.4. Analysis of Gender-Specific Differences within the Sub-Groups

In both fracture groups, the ap CTI did not show a significant difference between the two genders. Similar results were seen for the CCR. The lateral CTI of the male patients presenting with a femoral neck fracture was significantly higher than in female patients with the same fracture type (*p* = 0.022). Furthermore, in the non-fracture cohort, there was a significant difference in all measured parameters between male and female patients, with male subjects having a higher ap and lateral CTI (*p* = 0.031 and 0.030). The CCR was detected to be larger in female patients (*p* = 0.029). Relevant data are presented in Table 3.

### 3.5. Correlations between CTI and CCR within the Sub-Groups

**Figure 3:**
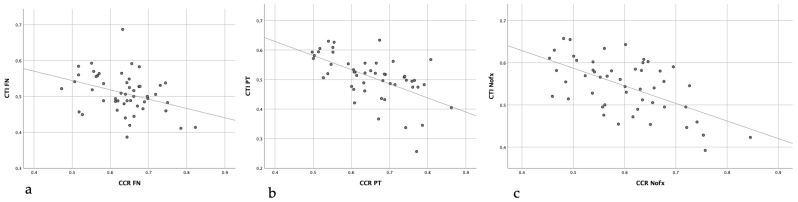
Correlation of CTI and CCR of the unaffected side in femoral neck fractures (**a**), trochanteric fractures (**b**) and the affected side in the non-injured femora (**c**).

The mean ap CTI of the fractured bone in the FNFx group showed a strong correlation with the mean CCR of the same hip (r = −0.683; *p* < 0.001). As depicted in Figure 3., in all three cohorts, the mean ap CTI of the uninjured side showed a moderate to strong correlation in comparison to the contralateral bone.

## 4. Discussion

The main finding of this study is that octogenarians to centenarians have a significantly lower CTI and CCR on the fractured proximal femur compared with the non-fractured control group. Furthermore, we were able to show that without a proximal femoral fracture, CTI and CCR showed no side-specific difference in geriatric patients. 

Several studies have shown a correlation between radiographic cortical parameters and bone quality [19,23,24]. In a study of the CTI and its correlation with the BMD, Sah et al. observed a significant difference between the CTI of osteoporotic bone (0.46 ± 0.09) and non-osteoporotic bone (0.55 ± 0.08; *p* = 0.008) [19]. Köse et al. have described similar values [23]. However, the mean age of these studies was relatively young and ranged between 64 and 72 years, which does not represent the most vulnerable age. A recent study by Ilyas et al. confirmed the correlation between reduced BMD and CTI, whereas they did not examine fractured proximal femora in a cohort of 156 patients with a mean age of 68 years [25]. Although the current mean age of patients with proximal femoral fractures is 75 years, the health system will be challenged by a growing population of even older patients in the upcoming years [26]. In this study, the mean age was 91 years and, therefore, the growing population of geriatric patients is represented more realistically. 

When looking at earlier published studies, heterogeneity in the analyzed side exists. Some authors infer conclusions by making a comparison between the unaffected femoral bone and the contralateral side [27]. Other publications did not provide detailed information on the measured side [28,29]. The current results, especially when focusing on the non-fractured cohort, show that CTI and CCR do not differ between the left and right proximal femurs. This allows us to infer the results from one side to another, at least in individuals without a fracture of the proximal femur. Furthermore, the observed ICC values for both measures were good to excellent. This confirms the previously described high level of reliability of the applied measurement, which underlines its feasibility for implementation in daily clinical practice regarding the prediction of proximal femoral fragility fractures [24,27]. Pothong et al. also recently presented their findings with the CTI being of a high predictive value for subsequent contralateral fragility hip fractures, underlining the importance of the CTI for daily clinical routines [30]. Since different and more detailed tools for fracture risk assessment were implemented in the past, the question could arise as to why use the CTI or CCR. Due to the lack of objectified BMD data through DEXA measurements or the limited availability of validated tools for all populations, their use might not be accessible for every clinician. In these situations, simple and reliable radiographic bone measurements could potentially be used. As described by Wang et al., who examined the correlation of fracture risk assessment tool (FRAX) scores with plain radiographic measurements for osteoporotic patients, lower ap and lateral CTI values reversely correlate with higher FRAX scores [18]. These findings could enable us to, for example, use the CTI to approximate the risk for fracture occurrence in the proximal femur. Therefore, these plain radiographic measures could be a valuable addition to the already implemented and reliable prediction methods such as the fracture risk assessment tool (FRAX) [31]. 

Looking at the gender-specific data, we were able to show that both CTI parameters as well as the CCR were significantly different for patients who did not suffer from a proximal femoral fracture. We hereby confirmed the previously described results by Nguyen et al., who analyzed non-fractured femora and showed a significant smaller CTI in female patients compared to male patients (*p* < 0.001) [32]. Interestingly, these gender-dependent results could not be displayed for patients with a fractured femoral bone, showing that scientific data on this issue are still lacking.

In addition to the obvious drawbacks of the retrospective design, certain limitations have to be addressed. At first, the differences in the results in the fracture groups could be influenced by the femoral rotational deviation of the standard patient’s positioning in the ap radiographs. Although it slight internal rotation of both legs should be aimed for, this might not be implemented in daily practice as long as affected patients suffer pain in a fracture situation. Another limitation of this study is the lack of an analysis and comparison of the radiographic parameters with additional fracture risk assessment tools, such as FRAX^®^ (Fracture Risk Assessment Tool), SCORE (Simple Calculated Osteoporosis Risk Estimation), QFracture^®^ and CAROC (Canadian Association of Radiologists and Osteoporosis Canada). Fracture risk prediction often involves the evaluation of multiple factors, including the patient’s age, history of previous fractures, gender, bone mineral density and other specific risk factors.

Despite the limitations, this study is an important contribution to the literature since we were able to show the link between two reliable radiological measurements and their importance for fracture prediction, especially for the growing cohort of geriatric patients. Future studies are needed to further evaluate the influence of X-ray timing and thus the effect of femoral rotation on the results of plain radiographic measurements.

## 5. Conclusions

Radiographic measurements using CTI and Dorr’s CCR are reliable measurements with a good correlation in geriatric patients. Both parameters are associated with increased fracture rates compared to the healthy, unfractured side. Therefore, even for geriatric patients, the CTI and CCR may help to predict fracture risk and enable practitioners to provide better advice to patients in their daily practice.

## Figures and Tables

**Table 1:** Patients’ demographic characteristics of the sub-groups.

Variable	Femoral Neck Fracture	Trochanteric Fracture	Non-Fracture
Number of Patients	50	50	50
Gender, *n* (%)			
female	36 (72)	42 (84)	31 (62)
male	14 (28)	8 (16)	19 (38)
Age	92.4 ± 2.4	91.7 ± 3.8	87.7 ± 4.7
Affected side, *n* (%)			
left	19 (38)	19 (38)	24 (48)
right	31 (62)	31 (62)	26 (52)

If not stated differently, data are presented as mean ± standard deviation.

**Table 2:** Results and comparison of the mean ap, lateral CTI and CCR measurements within the three sub-groups.

	Fractured/Measured	Non-Fractured	*p*-Value
ap CTI *			
FNFx	0.434 ± 0.08	0.509 ± 0.06	<0.001
TFx	0.451 ± 0.09	0.508 ± 0.08	<0.001
NFx	0.545 ± 0.06	0.548 ± 0.07	0.527
lateral CTI *			
FNFx	0.417 ± 0.07		
TFx	0.416 ± 0.06		
NFx	0.458 ± 0.08		
CCR *			
FNFx	0.735 ± 0.11	0.636 ± 0.08	<0.001
TFx	0.770 ± 0.17	0.653 ± 0.09	<0.001
NFx	0.6 ± 0.09	0.592 ± 0.1	0.291

ap = antero-posterior; CTI = cortical thickness index; CCR = canal calcar ratio; FNFx = femoral neck fracture; TFx = trochanteric fracture; NFx = non-fracture. * Data are presented as mean ± standard deviation. Significance was set at *p* < 0.05.

**Table 3:** Gender-specific results and comparison of the mean ap, lateral CTI and CCR measurements within the three sub-groups.

	Female	Male	*p*-Value
ap CTI *			
FNFx			
fractured	0.422 ± 0.08	0.463 ± 0.1	0.128
non-fractured	0.503 ± 0.05	0.525 ± 0.07	0.199
TFx			
fractured	0.453 ± 0.08	0.439 ± 0.09	0.290
NFx			
measured	0.53 ± 0.07	0.57 ± 0.05	0.031
contralateral	0.53 ± 0.07	0.57 ± 0.05	0.030
lateral CTI *			
FNFx	0.401 ± 0.06	0.457 ± 0.08	0.022
TFx	0.404 ± 0.06	0.451 ± 0.06	0.603
NFx	0.437 ± 0.08	0.491 ± 0.08	0.021
CCR *			
FNFx	0.741 ± 0.1	0.721 ± 0.1	0.554
TFx	0.656 ± 0.9	0.638 ± 0.1	0.653
NFx	0.622 ± 0.1	0.565 ± 0.7	**0.029**

ap = antero-posterior; CTI = cortical thickness index; CCR = canal calcar ratio; FNFx = femoral neck fracture; TFx = trochanteric fracture; NFx = non-fracture. * Data are presented as mean ± standard deviation. Significance was set at *p* < 0.05.

## Data Availability

Access to the data can be granted by the corresponding author upon request.
